# First Complete Genome Sequences of Porcine Bocavirus Strains from East Africa

**DOI:** 10.1128/genomeA.00093-17

**Published:** 2017-04-06

**Authors:** J. O. Amimo, J. Njuguna, E. Machuka, E. Okoth, A. Djikeng

**Affiliations:** a Department of Animal Production, Faculty of Veterinary Medicine, University of Nairobi, Nairobi, Kenya; b Biosciences of East and Central Africa—International Livestock Research Institute (BecA-ILRI) Hub, Nairobi, Kenya

## Abstract

Here, we report the first complete genome sequences of two strains of porcine bocavirus (JOA_011 and JOA_015) detected in Uganda and Kenya, respectively. These data will help in understanding the molecular and evolutionary characteristics of the porcine bocaviruses in this region and the development of appropriate diagnostic and control tools.

## GENOME ANNOUNCEMENT

Bocaviruses are nonenveloped single-stranded DNA (ssDNA) viruses 26 nm in diameter, with an approximately 5-kb genome. Bocaviruses have been detected in humans (1) and many animals (2), including canines (3), bovines (4), swine (5, 6), gorillas (7), and California sea lions (8). Bocaviruses have been recognized as potential emerging pathogens causing respiratory and gastrointestinal disease in domestic pigs. In this study, diverse strains of porcine bocaviruses (JOA_011 and JOA_015) were discovered and identified in piglet feces from a farm in Uganda and another farm in Kenya where the piglets had no clinical diarrhea. Viral DNA was purified from fecal samples, and sequencing was performed on an Illumina MiSeq for 300 cycles (150-bp paired-end reads). The reads were *de novo* assembled using Trinity version 2.0.6 (9), with the criteria of a 90% minimum overlap identity. BLASTx comparison of contigs with the NCBI nr protein database identified porcine bocavirus KU14 (GenBank accession no. KJ622366) as the most similar reference genome.

The genomes of JOA_011 and JOA_015 were ssDNA viruses comprising 5,200 bp and 5,184 bp, respectively, with a G+C content of 40%. Annotation was performed using the GATU software (10). Both genomes had three putative open reading frames (ORFs) encoding two nonstructural proteins (NS1, 1,908 nucleotides [nt]; NP1, 441 nt) and structural proteins (VP1/VP2, 1,851 nt). Phylogenetic analyses based on the full genomes and the ORFs indicated that the two strains were distantly related to most porcine bocaviruses (<78%), forming a distinct cluster within the genus *Bocavirus* together with H18 (GenBank accession no. HQ291308) and KU14 (GenBank accession no. KJ622366) detected in China and South Korea, respectively (11, 12). Sequence analysis showed that their genomes were closely related to each other (99.8%). They were also closely related to each other at their nonstructural proteins (NS1, 99.9%; NP1, 99.8%) and structural protein (VP1/2, 100%). The whole genomes (98.3%) and the three putative ORFs (NS1, 98.8%; NP1, 99.8 to 100%; VP1, 97.3%) of the two strains were closely related to the genome of the KU14 strain detected in South Korea; hence, they could be classified in the same genogroup. The whole genomes of the two strains were also related to that of the H18 strain (78%) detected in China; however, based on the International Committee on Taxonomy of Viruses (ICTV) species demarcation criteria of <95%, they could not be classified in the same genogroup with the H18 strain from China. The NS1, NP1, and VP1/2 genes of JOA_011 and JOA_015 shared <82%, <94%, and <79% sequence identity, respectively, with the nonstructural and structural genes of other porcine bocaviruses.

Under the existing criteria for the classification of bocaviruses by the ICTV, species are defined as having <95% homologous nonstructural gene DNA sequence (http://www.ictvdb.org/). Therefore, JOA_011 and JOA_015 strains should be classified as a prototype virus of a new porcine bocavirus species together with the KU14 strain from South Korea. These data will inform future investigations of evolutionary characteristics and molecular pathogenesis of bocaviruses in the swine populations in East Africa and the development of effective diagnostic tools and control strategies.

### Accession number(s).

The genome sequences of JOA_011 and JOA_015 strains were deposited in GenBank under accession numbers KY489985 and KY489986, respectively.
